# Therapeutic Effects of Tryptanthrin and Tryptanthrin-6-Oxime in Models of Rheumatoid Arthritis

**DOI:** 10.3389/fphar.2020.01145

**Published:** 2020-07-24

**Authors:** Liliya N. Kirpotina, Igor A. Schepetkin, Deepa Hammaker, Amanda Kuhs, Andrei I. Khlebnikov, Mark T. Quinn

**Affiliations:** ^1^ Department of Microbiology and Immunology, Montana State University, Bozeman, MT, United States; ^2^ Division of Rheumatology, Allergy, and Immunology, School of Medicine, University of California, San Diego, La Jolla, CA, United States; ^3^ Kizhner Research Center, Tomsk Polytechnic University, Tomsk, Russia; ^4^ Research Institute of Biological Medicine, Altai State University, Barnaul, Russia

**Keywords:** c-Jun N-terminal kinase, tryptanthrin, tryptanthrin-6-oxime, kinase inhibitor, arthritis, inflammation, collagen-induced arthritis, collagen antibody-induced arthritis

## Abstract

Rheumatoid arthritis (RA) is a chronic autoimmune disease involving joint and bone damage that is mediated in part by proteases and cytokines produced by synovial macrophages and fibroblast-like synoviocytes (FLS). Although current biological therapeutic strategies for RA have been effective in many cases, new classes of therapeutics are needed. We investigated anti-inflammatory properties of the natural alkaloid tryptanthrin (TRYP) and its synthetic derivative tryptanthrin-6-oxime (TRYP-Ox). Both TRYP and TRYP-Ox inhibited matrix metalloproteinase (MMP)-3 gene expression in interleukin (IL)-1β-stimulated primary human FLS, as well as IL-1β–induced secretion of MMP-1/3 by FLS and synovial SW982 cells and IL-6 by FLS, SW982 cells, human umbilical vein endothelial cells (HUVECs), and monocytic THP-1 cells, although TRYP-Ox was generally more effective and had no cytotoxicity *in vitro*. Evaluation of the therapeutic potential of TRYP and TRYP-Ox *in vivo* in murine arthritis models showed that both compounds significantly attenuated the development of collagen-induced arthritis (CIA) and collagen-antibody–induced arthritis (CAIA), with comparable efficacy. Collagen II (CII)-specific antibody levels were similarly reduced in TRYP- and TRYP-Ox-treated CIA mice. TRYP and TRYP-Ox also suppressed proinflammatory cytokine production by lymph node cells from CIA mice, with TRYP-Ox being more effective in inhibiting IL-17A, granulocyte-macrophage colony-stimulating factor (GM-CSF), and receptor activator of nuclear factor-κB ligand (RANKL). Thus, even though TRYP-Ox generally had a better *in vitro* profile, possibly due to its ability to inhibit c-Jun N-terminal kinase (JNK), both TRYP and TRYP-Ox were equally effective in inhibiting the clinical symptoms and damage associated with RA. Overall, TRYP and/or TRYP-Ox may represent potential new directions for the pursuit of novel treatments for RA.

## Introduction

Rheumatoid arthritis (RA) is an autoimmune disease that involves inflammation and progressive damage to distal joints, as well as inflammation and injury to other organs of the body (Firestein, 2003; Sharif et al., 2018). Thus, optimal therapeutic approaches should be developed to prevent inflammation, immune system dysregulation, and bone destruction associated with this disease, while still exhibiting enhanced safety and efficacy. Natural compounds have been considered as potential alternative or complementary treatments, as these compounds have been shown to possess a broader diversity in chemical space and, as a result, have significantly impacted drug development for many diseases (Hong, 2011). Indeed, many natural products have been shown to exhibit potential for treatment of inflammatory diseases (Lu et al., 2015) and have been evaluated in pre-clinical and clinical trials. For example, triptolide and its derivatives have been evaluated for their therapeutic effects in RA (Han et al., 2012; Tang and Zuo, 2012). Likewise, the plant-derived drug paclitaxel has been shown to inhibit collagen-induced arthritis (CIA) in mice (Xu et al., 2019).

Tryptanthrin (TRYP) (indolo[2,1-*b*]quinazolin-6,12-dione) is a well-known alkaloid and antibiotic that can be isolated from *Candida lypolica* (Brufani et al., 1971), higher plants (Bergman et al., 1985), and several species of marine micro- and macroorganisms [for review (Agafonova and Moskovkina, 2018)]. This compound has various pharmacological properties, including anti-inflammatory (Recio et al., 2006; Iwaki et al., 2011; Pathania et al., 2014), antimicrobial (Honda et al., 1979), antiviral (Tsai et al., 2020), and anti-tumor activities (Kimoto et al., 2001; Liao and Leung, 2013). For example, TRYP has been reported to reduce leukotriene-formation in human neutrophils and rat pleural exudates (Pergola et al., 2012). Likewise, TRYP was found to be effective in protecting mice against experimentally-induced colitis *via* regulation of the tumor necrosis factor (TNF)/nuclear factor (NF)-κB and interleukin (IL)-6/signal transducer and activator of transcription 3 (STAT3) signaling pathways (Wang et al., 2018). Although there are no reported studies regarding the effects of TRYP on RA, the signaling pathways impacted by TRYP clearly play roles in RA pathogenesis [e.g., see (Lubberts, 2015; Mitchell and Carmody, 2018)]. Thus, we hypothesized that TRYP or its structural analogs might be effective treatments for RA.

Structural modification of natural compounds can increase compound potency and selectivity, enhance their pharmacological properties, and significantly diminish their detrimental effects (Guo, 2017). Several TRYP derivatives with various tetracyclic scaffold modifications have been developed, including compounds with anti-plasmodium and anti-toxoplasma properties (Krivogorsky et al., 2008; Onambele et al., 2015), indoleamine 2,3-dioxygenase inhibitors (Yang et al., 2013), and DNA triplex stabilizing agents (Chen et al., 2007). Recently, we found that tryptanthrin-6-oxime (TRYP-Ox) had high affinity for JNK1-3 and also blocked activation of NF-κB/AP-1 and the production of IL-6 by lipopolysaccharide-treated monocytic cells (Schepetkin et al., 2019). Since JNK inhibition has potential for reducing inflammation associated with RA, it is reasonable that JNK inhibitors could be developed as RA therapeutics (Han et al., 2001; Bogoyevitch et al., 2010; Koch et al., 2015). Indeed, we found that 11*H*-indeno[1,2-*b*]quinoxalin-11-one oxime salt (**IQ-1S**) was an effective JNK inhibitor that blocked proinflammatory cytokine production and that **IQ-1S** treatment had a beneficial therapeutic effect in a mouse model of RA (Schepetkin et al., 2012; Schepetkin et al., 2015). Moreover, we also found that the indolo[2,1-*b*]quinazo-lin-6,12-dione (TRYP) structure was analogous to the 11*H*-indeno[1,2-*b*]quinoxalin-11-one scaffold (Schepetkin et al., 2019).

In the present study, we expanded our search for potential biological targets of TRYP and TRYP-Ox using a reverse pharmacophore mapping approach and again found that JNK1 and JNK3 were potential therapeutic targets of TRYP-Ox but not for TRYP. Furthermore, we evaluated anti-inflammatory effects of TRYP and TRYP-Ox *in vitro* and found that TRYP-Ox effectively inhibited IL-1β-induced IL-6 secretion by FLS, SW-982 synovial cells, and THP-1 monocytic cells, whereas TRYP was generally less effective. We also investigated the effect of these compounds *in vivo* using collagen-induced arthritis (CIA) and collagen-antibody-induced arthritis (CAIA) models of RA and found that TRYP-Ox significantly reduced the clinical symptoms and cartilage damage in CIA and CAIA. Surprisingly, TRYP was also effective in treating CIA and CAIA, although inhibition of cartilage destruction was more effective with TRYP-Ox. The therapeutic effects of TRYP and TRYP-Ox in CIA were associated with reduced levels of CII-specific antibodies and inhibition of proinflammatory cytokine production by lymph node (LN) cells. Overall, TRYP-Ox has a relatively greater therapeutic potential for treatment of RA compared to TRYP and represents a potential new direction for pursuit of novel treatments for RA.

## Materials and Methods

### Compounds

TRYP was purchased from Combi-Blocks (San Diego, CA, USA). TRYP-Ox and IQ-1S were synthesized, as described previously (Schepetkin et al., 2019). The JNK inhibitor SP600125 was from Tocris Bioscience (Ellisville, MO, USA). For *in vitro* studies, the compounds were dissolved in dimethyl sulfoxide (DMSO) and diluted into the desired buffer or culture media. For *in vivo* treatments, the compounds were suspended in sterile phosphate buffer saline (PBS).

### CIA Induction, Treatment, and Clinical Evaluation

DBA1/J male mice (6–8 weeks) were obtained from The Jackson Laboratories (Bar Harbor, ME, USA). All animal experiments were performed in accordance with National Institutes of Health guidelines and approved by the Montana State University Institutional Animal Care and Use Committee.

To induce CIA in DBA1/J mice, immunization-grade bovine Type II collagen (CII) (Chondrex, Redmond, WA, USA) was solubilized in 0.05 M acetic acid (2 mg/ml), and 100 μg of CII emulsified in complete Freund’s adjuvant containing 4 mg/ml *Mycobacterium tuberculosis* (Chondrex) were injected subcutaneously (*s.c*.) in the tail (Kochetkova et al., 2010). Using this method, nearly 100% of mice consistently showed clinical symptoms by day 25.

Suspensions of TRYP and TRYP-Ox or sterile saline were injected intraperitoneally (*i.p*.) at 30 mg/kg daily beginning on day 8 after the CII challenge, as indicated, and continued until day 42 post-CII challenge. Mice were scored using a scale of 0 to 3 for each limb, with a maximal total score of 12, as previously described (Schepetkin et al., 2015): 0, no signs of inflammation; 1, mild redness or swelling of single digits; 2, significant swelling of ankle or wrist with erythema; and 3, severe swelling and erythema of multiple joints.

### CAIA Induction, Treatment, and Clinical Evaluation

CAIA was induced in 7- to 8-week-old BALB/c mice from Charles River Laboratories (Wilmington, MA, USA) by *i.p*. injection of a cocktail of monoclonal anti-CII antibodies (Chondrex; 1.5 mg/mouse) on day 0, followed by *i.p*. lipopolysaccharide (LPS) injection (30 μg LPS from *Escherichia coli* strain 0111:B4 in PBS) on day 3. Control animals received an equal volume of PBS. Suspensions of TRYP, TRYP-Ox, and **IQ-1S** (all compounds in dose 30 mg/kg) or sterile saline were injected *i.p.* daily beginning at day 1 after injection of anti-CII Abs and continued until day 9.

Arthritis development was evaluated daily for 10 days post-injection of the monoclonal antibody cocktail. Arthritis symptoms were scored on scale of 0 to 4 in accordance with the Chondrex Mouse Arthritis Scoring System in a blinded manner, as follows: 0, no signs of inflammation; 1, mild redness or swelling of single joint (one of the interphalangeal joints, metacarpophalangeal joints, carpal joint for the front paw, and tarsal joint for the hind); 2, two joints have redness and swelling; 3, all three joint types have redness and swelling; 4, maximal redness and swelling of entire paw leads to the disappearance of anatomical definition.

### Histopathology

At 44 days after CII injection (CIA) or 14 days after anti-CII antibody injection (CAIA), the mice were euthanized, and their hind limbs were collected and fixed in 10% neutral buffered formalin. The limbs were decalcified in 5% formic acid for 3 to 6 days, and the joints were embedded in paraffin. Eight 8-μm sections were cut using a sagittal projection with 40 µm spacing of the cuts. Hematoxylin and eosin (H&E) and toluidine blue (TB) staining were performed for each sample.

H&E histopathological scores for the joints were determined by using a graded scale (Schramm et al., 2004), separately for cartilage destruction, pannus formation, and synovial cells changes. For cartilage destruction, the scale was: 0—normal, superficial zone is smooth; 1—superficial fibrillations, some loss of surface lamina; 2—vertical clefts/erosion to the calcified cartilage up to 25% of the surface; 3—vertical clefts/erosion to the calcified cartilage more than 25% of the surface, severe chondrocytes, and cartilage matrix loss with new bone tissue substitution, bone destruction. For pannus formation, the scale was: 0—normal; 1—exudate in the joint; 2—mild infiltration of the inflammatory cells; 3—heavy inflammatory infiltration and debris in the joint). For synovial changes, the scale was: 0—normal; 1—initial hyperplasia of the cells, with occasional infiltration of inflammatory cells; 2—focal infiltration of inflammatory cells into the synovial layers; 3—diffused infiltration of inflammatory cells into synovium. All sections of the hind paws and knee joints were examined, the highest score was recorded, with a total maximum score of 36 possible per mouse. The loss of the proteoglycans and cartilage degeneration was also scored in TB-stained sections on a graded scale of 0–3, as previously described (Bernotiene et al., 2004): 0—no cartilage loss; 1 – minimal chondrocytes and proteoglycan loss in superficial zone; 2—moderate chondrocytes and proteoglycan loss into middle zone but above tidemark; and 3—severe cartilage degeneration through tidemark, with the maximum score of 12 possible per mouse.

In CAIA, animals were sacrificed on day 14, and their forward and hind limbs were processed as described above. Three sections in the sagittal projection of joints, 40 µm apart, were examined by the same system with the grades ranging 0 to 4. Total H&E histopathological score for three sections included cartilage destruction, pannus formation, and synovial cells changes. For cartilage destruction, the scale was: 0—normal, superficial zone is smooth; 1—superficial fibrillations, some loss of surface lamina; 2—vertical clefts/erosion to the calcified cartilage up to 25% of the surface; 3—vertical clefts/erosion to the calcified cartilage 25% to 50% of the surface, 4—vertical clefts/erosion to the calcified cartilage more than 50% of the surface, severe chondrocytes, and cartilage matrix loss with new bone tissue substitution. For pannus formation the scale was: 0—normal; 1—exudate in the joint; 2—single inflammatory cells in the exudate; 3—mild infiltration of the inflammatory cells; 4—heavy inflammatory infiltration and debris in the joint. For synovial membrane changes the scale was: 0 – normal, 1 to 2 layer of synovial lining cells; 1—initial hyperplasia of the cells and increased number of lining cells layers; 2—increased number of lining cell layers, more than 3 to 4 layers and/or proliferation of sub-synovial tissue; 3—more than 4 layers of lining cells, focal infiltration of inflammatory cells, proliferation of subsynovial tissue; 4—more than four layers of lining cells, diffused infiltration of inflammatory cells, proliferation of sub-synovial tissue.

### Anti-CII Antibody Enzyme-Linked Immunosorbent Assay (ELISA)

Serum samples from mice with CIA were collected on day 44 after CII injection. Samples of the diluted sera were added to microtiter plates (Greiner Bio-One, Monroe, NC, USA) coated with 2 μg/ml ELISA grade mouse CII (Chondrex). Horseradish peroxidase-conjugated goat anti-bovine total IgG, IgG1, IgG2a, IgG2b, and IgG3 (Southern Biotechnology Associates, Birmingham, AL, USA) were used for detection. The samples were developed with 2,2’-azino-bis(3-ethylbenzothiazoline-6-sulphonic acid (Moss Inc., Pasadena, MD, USA), and absorbance was monitored at 415 nm using a SpectraMax Plus microplate reader. Endpoint titer represents reciprocal log 2 for the serum dilution with absorbance ≥ 0.1 above negative control.

### Determination of Cytokines in LN Supernatants

At the end of the study (day 44), we isolated the axillary, inguinal, and popliteal LN and purified mononuclear cells from the LN. These cells were cultured in 6-well plates (5 × 10^6^ cells/ml) without or with 50 μg/ml CII for 3 days in RPMI-1640 medium supplemented with 10% of fetal bovine serum (FBS) (Invitrogen, Carlsbad, CA, USA), 2 mM l-glutamine, 100 U/ml penicillin, 100 μg/ml streptomycin, 1 mM sodium pyruvate, and 0.1 mM nonessential amino acids (Kochetkova et al., 2008). The levels of cytokines, including tumor necrosis factor (TNF), granulocyte-macrophage colony-stimulating factor (GM-CSF), IL-1β, IL-5, IL-6, IL-10, IL-17A, and receptor activator of nuclear factor-κB ligand (RANKL) were measured in LN culture supernatants using ELISA kits (BD Biosciences, San Jose, CA, USA) for mouse cytokines.

### Cell Lines

The SW982 human synovial cell line was obtained from the America Type Culture Collection (ATCC, Rockville, MD, USA). SW982 cells were maintained in ATCC-formulated Leibovitz’s L-15 Medium supplemented with 10% FBS, 100 U/ml penicillin, and 100 µg/ml streptomycin. Human monocytic THP-1 cells (ATCC) were cultured in RPMI-1640 medium (Mediatech Inc., Herndon, VA, USA) supplemented with 10% (v/v) FBS, 100 U/ml penicillin, and 100 µg/ml streptomycin. Human umbilical vein endothelial cells (HUVECs) (ATCC, Rockville, MD, USA) were cultured in vascular cell basal medium (ATCC) supplemented with components from the endothelial cell growth kit-VEGF (ATCC): 2% FBS, 10 mM L-glutamine, 5 ng/ml recombinant human epidermal growth factor (rh EGF), 5 ng/ml recombinant human vascular endothelial growth factor (rh VEGF), 15 ng/ml recombinant human insulin-like growth factor (rh IGF-1), 1 μg/ml hydrocortisone hemisuccinate, 0.75 U/ml heparin sulfate, 50 μg/ml ascorbic acid, 100 U/ml penicillin, and 100 μg/ml streptomycin according to the manufacturer’s specifications. All cell cultures were maintained in polystyrene tissue culture flasks at 37°C and 5% CO_2_.

### Isolation and Culture of Primary Fibroblast-Like Synoviocytes

This study was approved by the Institutional Review Board of University of California, San Diego School of Medicine (La Jolla, CA, USA). Informed consent was obtained from all participants. Anonymous synovial tissue samples were obtained from six patients with RA at the time of total joint replacement (5 females, 1 male; age range, 44–72 years), as previously described (Alvaro-Gracia et al., 1990). The diagnosis of RA conformed to American College of Rheumatology 1987 revised criteria (Arnett et al., 1988). The synovium was minced and incubated with collagenase type VIII (0.5 mg/ml) (Sigma-Aldrich) in serum-free RPMI-1640 (Life Technologies, Grand Island, NY, USA) for 2 h at 37°C, filtered, extensively washed, and cultured in DMEM (Life Technologies) supplemented with 10% FBS (Gemini Bio Products, Calabasas, CA, USA), penicillin, streptomycin, gentamicin, and glutamine in a humidified 5% CO_2_ atmosphere. Cells were allowed to adhere overnight, nonadherent cells were removed, and adherent fibroblast-like synoviocytes (FLS) were split at 1:3 when 70%–80% confluent. FLS were used from passages 3 through 9, during which time they are a homogeneous population of cells (<1% CD11b positive, <1% phagocytic, and <1% Fc*γ*RII and Fc*γ*RIII receptor positive).

### Analysis of Matrix Metalloproteinase (*MMP)* Expression in FLS

For *MMP* mRNA analysis, FLS were plated in six-well plates, cultured until 80% confluence, and subsequently serum starved (1% FBS/DMEM) for 24 h. The cells were treated with TRYP or TRYP-Ox (4, 10, and 25 μM) or vehicle (DMSO) for 1 h before IL-1β stimulation (2 ng/ml) for 6 h. The mRNA was isolated and reverse transcribed to obtain cDNA. Quantitative real-time polymerase chain reaction was performed using primer probe sets for *MMP3* and glyceraldehyde 3-phosphate dehydrogenase (*GAPDH*) (Life Technologies). Threshold cycle values were obtained and normalized to *GAPDH* expression.

### Analysis of c-Jun Phosphorylation in FLS

Cultured FLS (passage 4) were plated at 2 × 10^5^ cells in six-well plates overnight in DMEM containing 10% FBS and synchronized for 24 h in DMEM/0.1% FBS. The cells were pretreated for 1 h with different concentrations of TRYP, TRYP-Ox, or DMSO (vehicle control) and then stimulated with 2 ng/ml IL-1β or medium for 15 min at 37°C. The cells were then washed once with ice-cold PBS and lysed with modified radioimmunoprecipitation assay buffer [50 mM HEPES, pH 7.4, 150 mM NaCl, 1% Triton X-100, 10% glycerol, 2.5 mM MgCl_2_, 1.0 mM EDTA, 20 mM *β*-glycerophosphate, 10 mM NaF, 1 mM Na_3_VO_4_, and Protease inhibitor cocktail (Roche, Indianapolis, IN)]. Protein concentration of the lysates was measured using a MicroBCA Assay kit (Pierce, Rockford, IL), and 40 *μ*g lysate was subjected to 10% SDS-PAGE and Western blot analysis. Anti–phospho-c-Jun (Ser63) was purchased from Cell Signaling Technology (Danvers, MA), and anti-mouse GAPDH was from Santa Cruz Biotechnology (Santa Cruz, CA).

### Determination of MMP-1/3 and IL-6 in Cell Culture Supernatants

FLS, HUVEC, SW982, and THP-1 cells were plated in 96 well plates (10^4^ to 10^5^ cells/well) for 48 h before treatment. At 2 days post-confluency, the cells were serum-starved and washed twice (with the exception of THP-1 cell, which were not washed) with FBS-free medium prior to the addition of test compounds. The cells were incubated in the presence or absence of test compounds or DMSO (vehicle control) for 30 min at 37°C in a 5% CO_2_ atmosphere, IL-1β (5 ng/ml) was added, and the cells were incubated for an additional 24 h. Culture supernatants were collected and stored at –80°C. The levels of MMP-1 and MMP-3 were determined in culture supernatants using commercially available ELISA kits according to the manufacturer’s instructions (R&D System, Minneapolis, MN, USA). IL-6 levels were also determined in the supernatants using a human IL-6 ELISA kit (BD Biosciences, San Jose, CA, USA).

### AChE Inhibition Assay

The inhibitory effect of test compounds on AChE activity was performed using an AChE inhibitor screening kit from Sigma-Aldrich. The kit is based on an improved Ellman method, whereby thiocholine produced from AChE activity forms a yellow color with 5,5’-dithiobis(2-nitrobenzoic acid), and the intensity the color (412 nm) is proportional to enzyme activity. The concentration of compound required to cause 30% inhibition (IC_30_) was used. IC_30_ was obtained by graphing the % inhibition of enzyme activity versus the logarithm of concentration of test compound using 5–7 tested concentrations.

### Cytotoxicity Assay

Cytotoxicity was analyzed using a CellTiter-Glo Luminescent Cell Viability Assay Kit (Promega), according to the manufacturer’s protocol. Briefly, cells were cultured at a density of 10^4^ to 10^5^ cells/well with different concentrations of test compounds (final concentration of DMSO was 1%) for the indicated periods of time (2, 12, or 24 h) at 37°C and 5% CO_2_. Following treatment, substrate was added to the cells, and the samples were analyzed with a Fluoroscan Ascent FL microplate reader.

### PharmMapper Target Identification

The protein targets of TRYP and TRYP-Ox were analyzed using PharmMapper (Liu et al., 2010). PharmMapper is a tool that identifies potential targets for a given small molecule using an “invert” pharmacophore mapping procedure. PharmMapper utilizes reference databases of protein drug targets encoded by sets of pharmacophore points for faster mapping. Initial structures of the test compounds were prepared using ChemDraw 16.0 software and saved in Tripos MOL2 format. The MOL2 files of TRYP and *Z*- and *E*-isomers of TRYP-Ox were uploaded into the PharmMapper server with automatic generation of up to 300 conformers for each compound switched on. We selected the “Human Protein Targets Only” database containing 2241 targets for pharmacophore mapping. The top 250 potential targets were retrieved and sorted by normalized fit score value.

The physicochemical properties of selected compounds were computed using SwissADME (http://www.swissadme.ch) (Daina et al., 2017).

### Statistical Analysis

A nonparametric Mann–Whitney *U* test was used for statistical analysis of CIA and CAIA clinical scores, histology scores, and cartilage destruction. All other data were analyzed by ANOVA with no correction, and differences were considered statistically significant if *p*<0.05.

## Results

### Identification of Potential Therapeutic Targets for TRYP and TRYP-Ox

Previously we used kinase profiling to demonstrate that TRYP-Ox had high binding affinity for JNK1-3, whereas TRYP had very low affinity for JNK1 and had no binding affinity for JNK2 and JNK3 (Schepetkin et al., 2019). To expand and enhance our understanding of the potential biological targets of TRYP/TRYP-Ox, we performed reverse pharmacophore mapping on TRYP and the *Z*- and *E*-isomers of TRYP-Ox using PharmMapper. This program compared a database of pharmacophore patterns with our test compounds and generated target information, including normalized fitness scores and pharmacophoric characteristics. As shown in **Table 1**, PharmMapper analysis indicated that the top three ranked potential targets for the *Z*- and *E*-isomers of TRYP-Ox were JNK1, JNK3, and complement factor B (CFB), confirming the high binding affinity for JNK1 and JNK3 that we found using kinase scanning and verifying that these are indeed relevant therapeutic targets for TRYP-Ox. In contrast, PharmMapper analysis of the parent alkaloid TRYP showed relatively low fitness scores for JNK1 and JNK3, with the top three ranked targets for TRYP being acetylcholinesterase (AChE), carboxylesterase 1 (CES-1), and transthyretin (TTR) (**Table 1**). The low fitness for TRYP binding to JNK is consistent with our previous kinase scanning studies (Schepetkin et al., 2019).

**Table 1:** Potential protein targets of TRYP and E/Z isomers of TRYP-Ox.

Compound	Rank	PDB ID	Fit score	Target name*
TRYP	**1**	1XLV	0.7668	**AChE**
	**2**	1YA8	0.7459	**CES-1**
	**3**	1E5A	0.7456	**TTR**
	36	2H96	0.5262	JNK1
	47	3FV8	0.4908	JNK3
	>146	–	–	CFB
(*E*)TRYP-Ox	**1**	1UKI	0.9836	**JNK1**
	**2**	1PMV	0.964	**JNK3**
	**3**	1RS0	0.9423	**CFB**
	4	1DVZ	0.9349	TTR
	6	1YA8	0.8217	CES-1
	18	1XLV	0.7108	AChE
(*Z*)TRYP-Ox	**1**	1UKI	0.9836	**JNK1**
	**2**	1PMV	0.964	**JNK3**
	**3**	1RS0	0.9423	**CFB**
	7	1YA8	0.7565	CES-1
	11	1E5A	0.7242	TTR
	>172	–	–	AChE

*AChE, acetylcholinesterase; CES-1, carboxylesterase 1; TTR, transthyretin; CFB, complement factor B; JNK, c-Jun N-terminal kinase. The top 3 targets for each compound are highlighted in bold.

Since AChE was found to be the top target for TRYP and was also on the target list for TRYP-Ox, although not close to the top targets, we evaluated the direct AChE inhibitory effects of both TRYP and TRYP-Ox using an enzymatic test-system but found that both compounds had a relatively low anti-AChE activity, with TRYP being somewhat more active (**Table 2**). Thus, PharmMapper analysis supported the role of JNK1 and JNK3 as targets for TRYP-Ox, which is relevant to therapeutic intervention in RA, whereas no obvious targets related to RA intervention were found for TRYP.

**Table 2:** Effect of TRYP and TRYP-Ox on the top ranked molecular targets.

Compound	JNK1	JNK3	AChE inhibitionIC_30_ (µM)
	K_d_ (µM)^a^		
TRYP	23.0	No binding	4.2 ± 1.2
(*E*/*Z*)-TRYP-Ox	0.150	0.275	12.0 ± 3.9

^a^Previously reported binding affinity (Schepetkin et al., 2019).

### *In Silico* ADME Predictions

**Figure 1:**
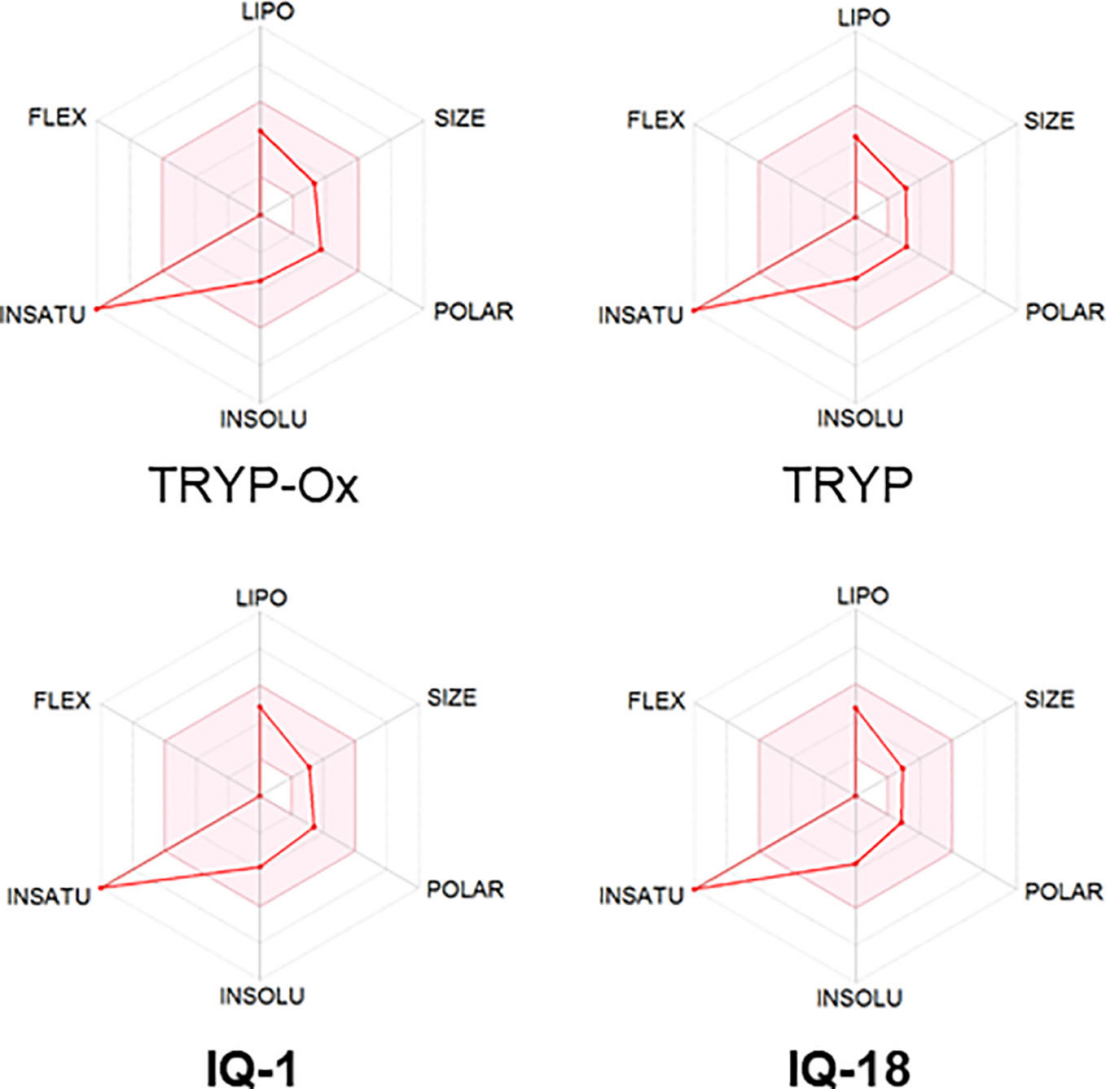
Bioavailability radar plots of JNK inhibitors TRYP-Ox and **IQ-1** and their ketone precursors TRYP and **IQ-18**. The plots depict the LIPO (lipophilicity), SIZE (molecular weight), POLAR (polarity), INSOLU (insolubility), INSATU (unsaturation), and FLEX (rotatable bond flexibility) parameters.

The ADME properties determining either the access of a potential drug to the target or its elimination by the organism are necessary during initial stages of drug discovery (Doogue and Polasek, 2013). TRYP and TRYP-Ox were analyzed for the most important physicochemical properties in comparison with indenoquinoxaline analogs **IQ-1** (an oxime formed after spontaneous hydrolysis of the JNK inhibitor **IQ-1S**) and **IQ-18** (the inactive ketone precursor of **IQ-1**) using the SwissADME tool (Daina et al., 2017). We found that these compounds were similar regarding many ADME properties (**Table 3**). Nevertheless, they differed markedly in the number of H-bond acceptors and donors, which we showed previously affected JNK binding affinities and locations within the JNK binding site for (Schepetkin et al., 2012; Schepetkin et al., 2015). We also created bioavailability radar plots that display an assessment of the drug-likeness of each compound. Six physicochemical properties are considered: lipophilicity, size, polarity, solubility, flexibility, and saturation. The physicochemical range on each axis is depicted as a pink area in which the radar plot of the molecule has to fall entirely to be considered drug-like (Daina et al., 2017). We found that all four compounds had similar bioavailability radar plots (**Figure 1**). Moreover, the charge distributions for **IQ-1** and TRYP-Ox were very similar, which is not surprising based on their similar structures (Schepetkin et al., 2019).

**Table 3:** Chemical structures and physicochemical properties of JNK inhibitors TRYP-Ox and IQ-1 and their ketone precursors TRYP and IQ-18.

	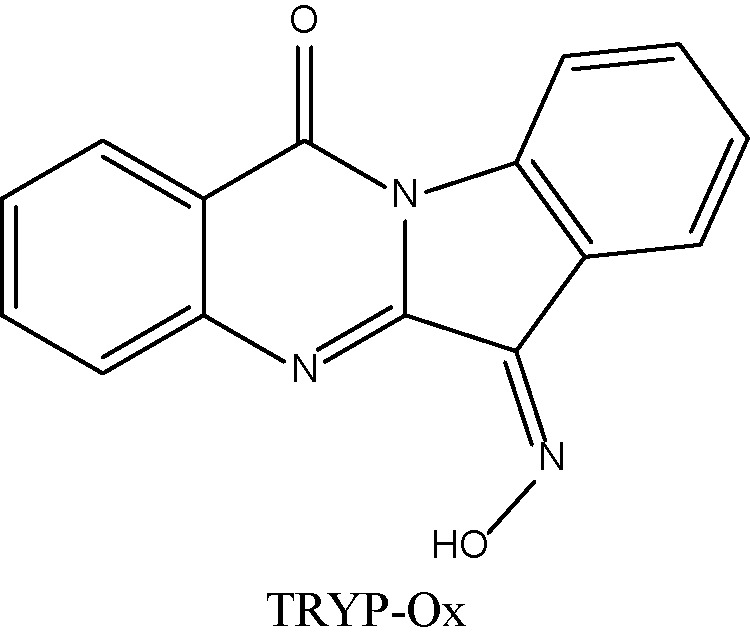 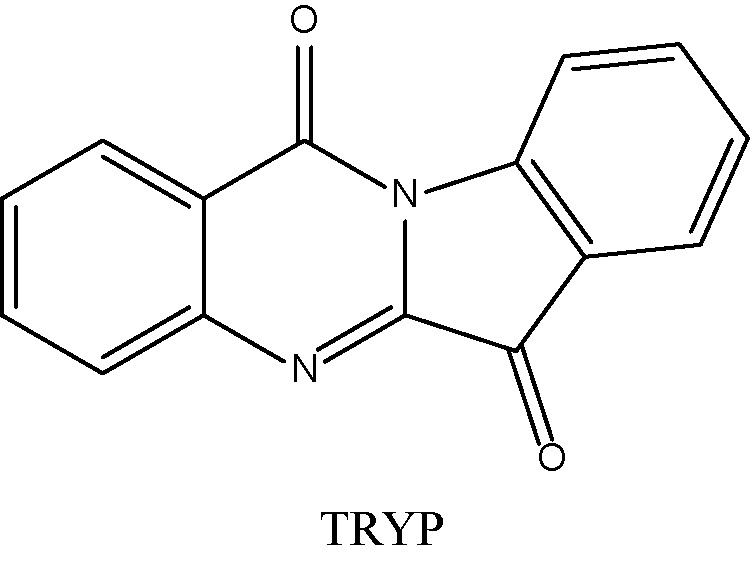 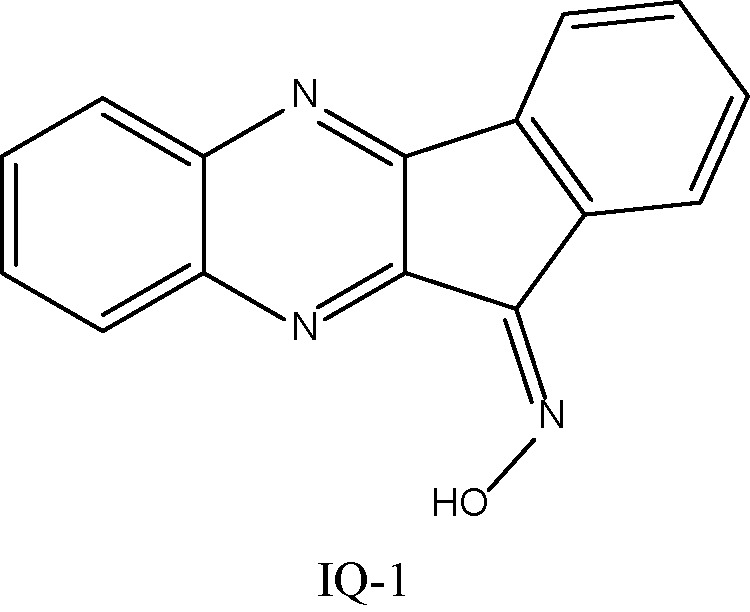 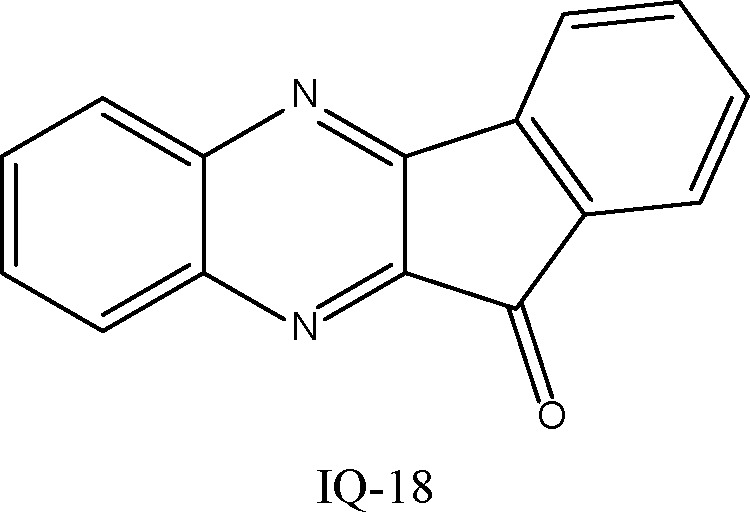			
Property	Compounds			
	TRYP	TRYP-Ox	IQ-1	IQ-18
Formula	C_15_H_8_N_2_O_2_	C_15_H_9_N_3_O_2_	C_15_H_9_N_3_O	C_15_H_8_N_2_O
M.W.	248.24	263.25	247.25	232.24
Heavy atoms	19	20	19	18
Fraction Csp^3^	0	0	0	0
Rotatable bonds	0	0	0	0
H-bond acceptors	3	4	4	3
H-bond donors	0	1	1	0
MR	70.77	74.77	72.4	68.41
tPSA	51.96	67.48	58.37	42.85
Log P	2.16	2.01	2.52	2.57

Log P, lipophilicity (consensus Log Po/w); M.W., molecular weight (g/mol); MR, molar refractivity; tPSA, topological polar surface area (Å^2^).

### TRYP and TRYP-Ox Inhibit *MMP3* Gene Expression and MMP1/3 and IL-6 Secretion

**Figure 2:**
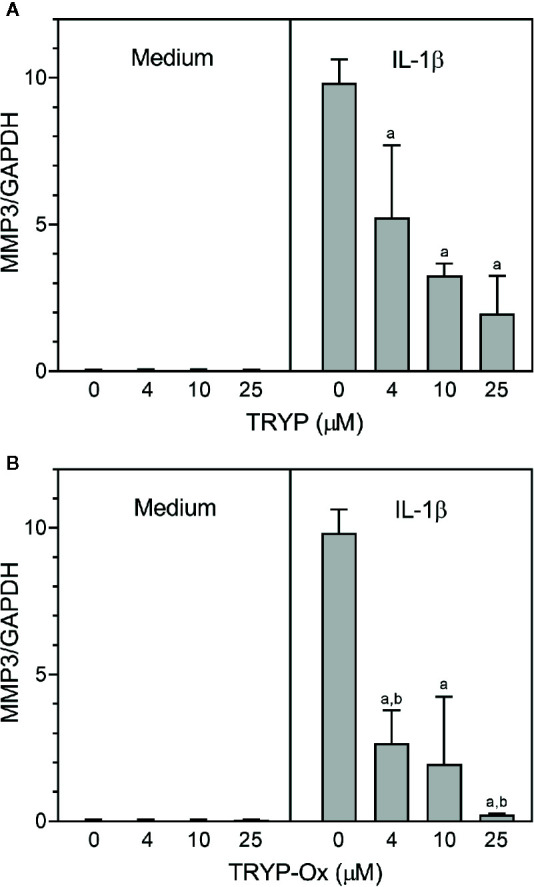
TRYP and TRYP-Ox decrease *MMP3* gene expression in FLS from RA patients. FLS were treated with the indicated concentrations of TRYP (Panel **A**) or TRYP-Ox (Panel **B**) and stimulated with control medium or IL-1β (2 ng/ml) for 6 h. *MMP3* gene expression was evaluated by quantitative polymerase chain reaction and normalized to glyceraldehyde 3-phosphate dehydrogenase (*GAPDH*), as described under *Materials and Methods*. The results are shown as the mean ± S.D. of three different RA lines. ^a^ *p *< 0.05 compared with the DMSO control; ^b^ *p* < 0.05 compared with TRYP.

**Figure 3:**
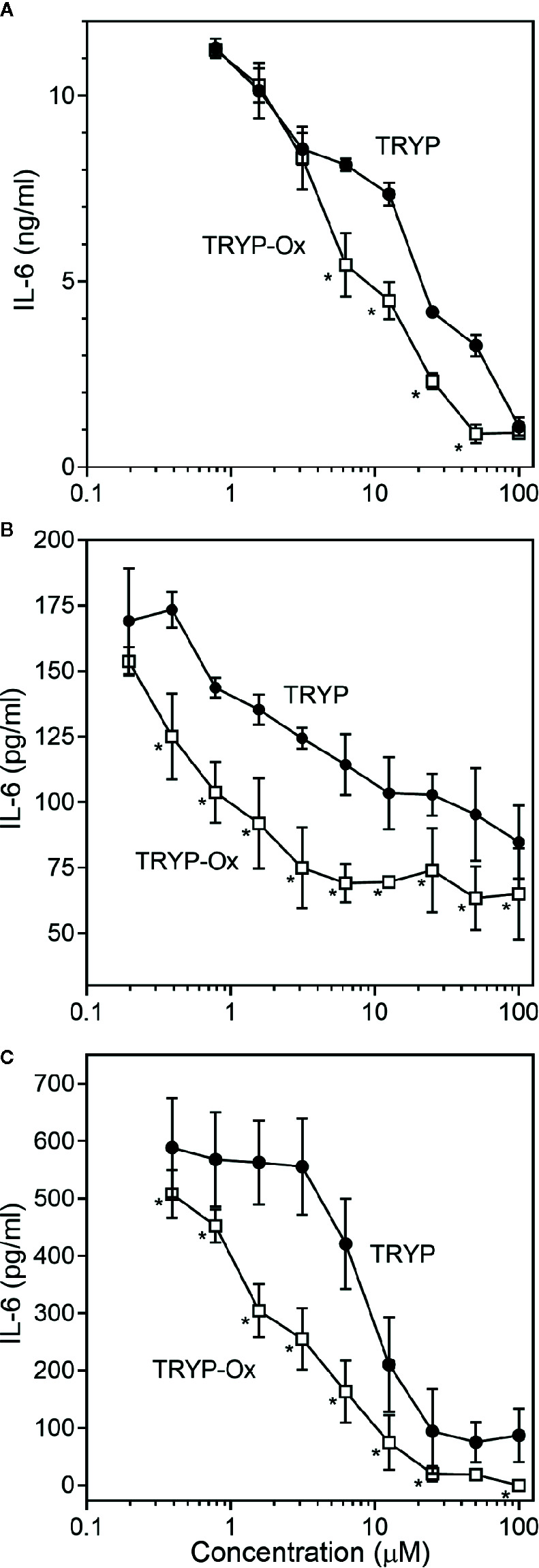
Effects of TRYP and TRYP-Ox on IL-1β-induced IL-6 secretion by FLS, SW982 cells, and HUVECs. FLS **(A)**, SW982 cells **(B)**, and HUVECs **(C)** were treated with the indicated compounds or DMSO for 30 min, followed by stimulation with 5 ng/ml IL-1β for 24 h. IL-6 levels were measured by ELISA. Values are expressed as mean ± S.D. from one experiment that is representative of 3 independent experiments. ^*^ *p*<0.05 compared with TRYP.

FLS obtained by arthroplasty from tissue of patients with RA are an excellent *in vitro* model for screening novel compounds with antiarthritic potential (Bartok and Firestein, 2010). We evaluated whether TRYP and TRYP-Ox altered *MMP3* expression by IL-1β–stimulated human FLS and found that cells pretreated with either of these compounds exhibited a dose-dependent reduction in *MMP3* mRNA levels compared with the vehicle-treated group, but that TRYP-Ox was significantly more active than TRYP (**Figure 2**). Numerous studies suggest that human SW982 synovial sarcoma cells share similar physiological and immunological properties with primary FLS and that IL-1β stimulation of SW982 cells can mimic the inflammatory status of synovial cells typically seen in RA patients (Tsuji et al., 1999; Kim et al., 2008; Chang et al., 2014). Thus, SW982 cells can be useful in screening of potential antiarthritic compounds, as FLS are in limited supply. Indeed, we found that TRYP and TRYP-Ox inhibited IL-1β–induced IL-6 secretion by FLS and SW982 cells, as well as by HUVECs and THP-1 monocytic cells, in a dose-dependent manner, with the most potent being TRYP-Ox (**Table 4A**). These compounds also inhibited IL-1β–induced MMP-1/3 secretion by FLS, SW982 cells, and HUVECs, with the most potent being TRYP-Ox in FLS and SW982 cells (**Table 4A**). As examples, the dose-dependent effects of TRYP and TRYP-Ox on IL-1β–induced secretion of IL-6 by FLS, SW982 cells, and HUVECs are shown in **Figure 3**. Note that TRYP-Ox was significantly more effective than TRYP in inhibiting IL-6 production in all three of these cell types.

**Table 4A:** Inhibitory effect of TRYP and TRYP-Ox on IL-1β-induced IL-6 and MMP-1/3 secretion by monocytic THP-1 cells, synovial SW982 cells, and HUVEC.

Compound	THP-1	SW982	HUVEC	FLS	SW-982	HUVEC	FLS	SW982	HUVEC	FLS
	IL-6				MMP-1			MMP-3		
	IC_50_ (µM)									
TRYP	9.3 ± 2.7	2.5 ± 1.1	6.4 ± 2.7	14.8 ± 1.8	7.8 ± 2.0	3.4 ± 1.1	1.2 ± 0.4	8.7 ± 0.3	12.8 ± 4.4	12.7 ± 4.6
TRYP-Ox	0.3 ± 0.12^#^	0.4 ± 0.2^#^	0.9 ± 0.3^#^	7.4 ± 1.2^#^	2.6 ± 0.8^#^	2.9 ± 0.1	0.7 ± 0.1	1.4 ± 0.1^#^	15.9 ± 5.8	3.8 ± 0.4^#^

The cells were treated with compounds for 30 min and then activated with 5 ng/ml IL-1β. After 24 h incubation, MMP-1/3 and IL-6 were evaluated in the supernatants by ELISA. IC_50_ values are presented as the mean ± S.D. of three to four independent experiments. ^#^p < 0.05 compared with TRYP.

To ensure that the effect of our compounds on functional activity *in vitro* were not affected by possible toxicity, we evaluated cytotoxicity of TRYP and TRYP-Ox at various concentrations (up to 50 µM) in all cultured cells (**Table 4B**). While TRYP-Ox exhibited no cytotoxicity in all of the cells tested, TRYP exhibited some cytotoxic effects, albeit at higher concentrations that those required to inhibit IL-6 and MMP-1/3 production. Thus, TRYP cytotoxicity may have some contribution to the inhibition of pro-inflammatory cytokine/enzyme production by some cell lines.

**Table 4B:** Cytotoxic effect of TRYP and TRYP-Ox on THP-1 cells, SW982 cells, HUVECs, and FLS.

Compound	THP-1	SW982	HUVEC	FLS
	IC_50_ (µM)			
TRYP	18.2 ± 2.3	19.1 ± 4.9	13.8 ± 1.5	16.7 ± 1.6
TRYP-Ox	N.T.	N.T.	N.T.	N.T.

N.T., no toxicity was observed at compound concentrations up to 50 µM.

**Figure 4:**
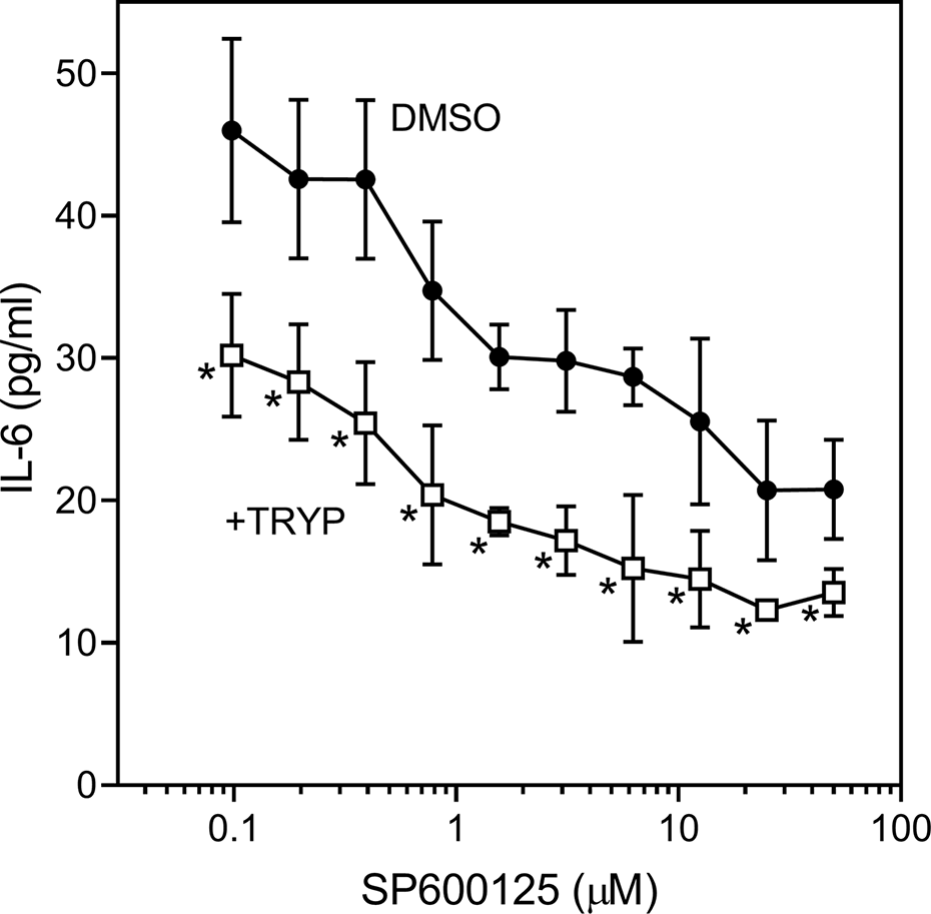
Effects of JNK inhibitor SP600125 and TRYP on IL-1β-induced IL-6 secretion by SW982 cells. Cells were treated with 5 µM TRYP or DMSO combined with the indicated concentrations of SP600125 for 30 min, followed by stimulation with 5 ng/ml IL-1β for 10 h. IL-6 levels were measured by ELISA. Values are expressed as mean ± S.D. of one experiment that is representative of 2 independent experiments. ^*^ *p* < 0.05 compared with SP600125 treatment alone.

We also evaluated the effects of SP600125, a commonly used JNK inhibitor (Bennett et al., 2001), on IL-6 secretion in SW982 synovial cells. Since SP600125 increased cytotoxicity of TRYP during a 24 h incubation, we used a shorter incubation period (12 h) that did not affect cell viability. We found that SP600125 dose-dependently inhibited IL-1β–induced IL-6 secretion by SW982 cells (IC_50_ = 1.3 ± 0.5 µM). In addition, combined treatment of SP600125 plus TRYP was evaluated. In the presence of 5 µM TRYP, the IC_50_ value for SP600125 was the similar to that observed when TRYP was absent (1.2 ± 0.2 µM), although TRYP acted synergistically with SP00125 to significantly increase the overall inhibition compared to SP00125 alone (**Figure 4**). Thus, TRYP and SP600125 may inhibit IL-6 production through different pathways.

### TRYP-Ox Inhibits c-Jun Phosphorylation in Rheumatoid FLS

**Figure 5:**
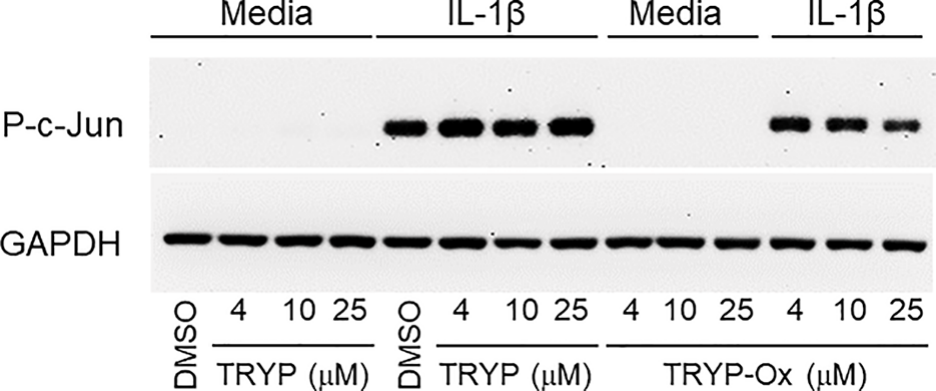
TRYP-Ox inhibits c-Jun phosphorylation induced by IL-1β in FLS from RA patients. FLS were pretreated for 1 h with the indicated concentrations of TRYP-Ox, TRYP, or DMSO and then stimulated with 2 ng/ml IL-1β or medium for 15 min at 37°C. Equal concentrations of cell lysates were separated by SDS-PAGE, followed by Western blotting for phospho-c-Jun (P-c-Jun) and GAPDH, as described. The blot shown is from one experiment that is representative of 2 independent experiments.

Previously, we showed that the JNK inhibitor **IQ-1S**, which is a close chemical analog of TRYP-Ox (see **Table 3**), inhibited c-Jun phosphorylation in FLS (Schepetkin et al., 2015). Analysis of the effects of TRYP and TRYP-Ox on c-Jun phosphorylation in IL-1β–stimulated FLS showed that TRYP-Ox also inhibited c-Jun phosphorylation, whereas TRYP had no effect (**Figure 5**), confirming our previous conclusion that TRYP-Ox, but not TRYP, is a JNK inhibitor (Schepetkin et al., 2019). Note that neither TRYP nor TRYP-Ox affected FLS viability during the 90-min period.

### TRYP and TRYP-Ox Reduce CIA Severity

Previously, we evaluated a range of **IQ-1S** doses (5, 20, 30, and 50 mg/kg *i.p*.) and found that 5 mg/kg was not very effective, whereas 30 and 50 mg/kg were equally effective in reducing arthritis in the CIA model (Schepetkin et al., 2015). Preclinical pharmacokinetic studies in rats demonstrated that after *i.p.* administration of **IQ-1S**, a rapid rise in the serum concentration was observed, peaking at 5 min, and the half-life in circulation was 12 h (Plotnikov et al., 2020). When mice were dosed with 30 mg/kg IQ-1S *i.p*., the serum exposure of the compound was also good, with AUC 0–12 h values of 7.4 µM/h (Schepetkin et al., 2012). Thus, based on the similarity in physicochemical properties of TRYP-Ox and **IQ-1S**, their high JNK binding affinity, the effectiveness of 30 mg/kg **IQ-1S** in previous CIA experiments, and the known preclinical pharmacokinetic studies for **IQ-1S**, we selected a 30 mg/kg dose for TRYP-Ox and TRYP treatment in our *in vivo* studies.

**Figure 6:**
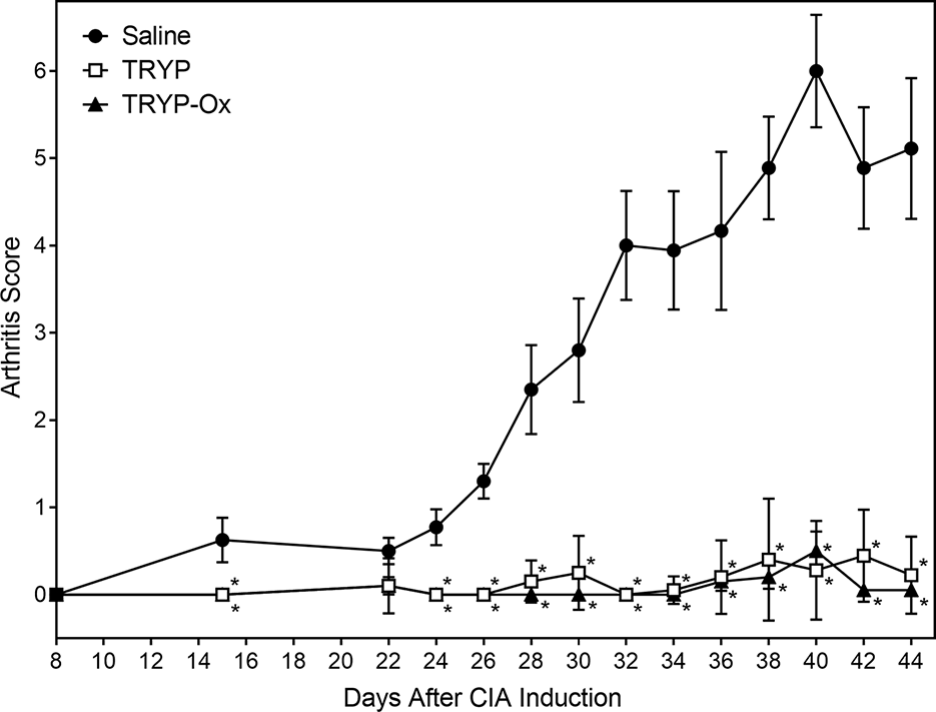
Therapeutic effects of TRYP and TRYP-Ox in CIA mice. Starting at day 8 after CII challenge, mice were treated *i.p.* daily with 30 mg/kg TRYP (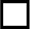) or 30 mg/kg (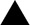) TRYP-Ox until day 42, and clinical scores were determined, as described. Control mice were treated *i.p.* daily with saline (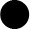). Results are shown as the mean of 10 mice per group ± S.D. ^*^ *p* < 0.05 compared with saline control.

CIA was induced in arthritis-susceptible DBA1/J mice by immunization with bovine CII emulsified in complete Freund’s adjuvant. To test the therapeutic potential of TRYP and TRYP-Ox on CIA, treatment was initiated at day 8 after CII challenge. Although disease symptoms appear usually at day 21 post-induction, immune pathologic processes begin soon after CII injection (Brand et al., 2003). Thus, we consider administration of compounds between days 0 and 21 following CIA initiation to be a combined prevention/treatment model. We found that mice treated *i.p.* daily with 30 mg/kg of TRYP or TRYP-Ox displayed significant reductions in CIA symptoms compared with vehicle-treated mice (**Figure 6**). Both TRYP and TRYP-Ox seem to be equally effective treatments for reducing clinical scores in the CIA model.

**Figure 7:**
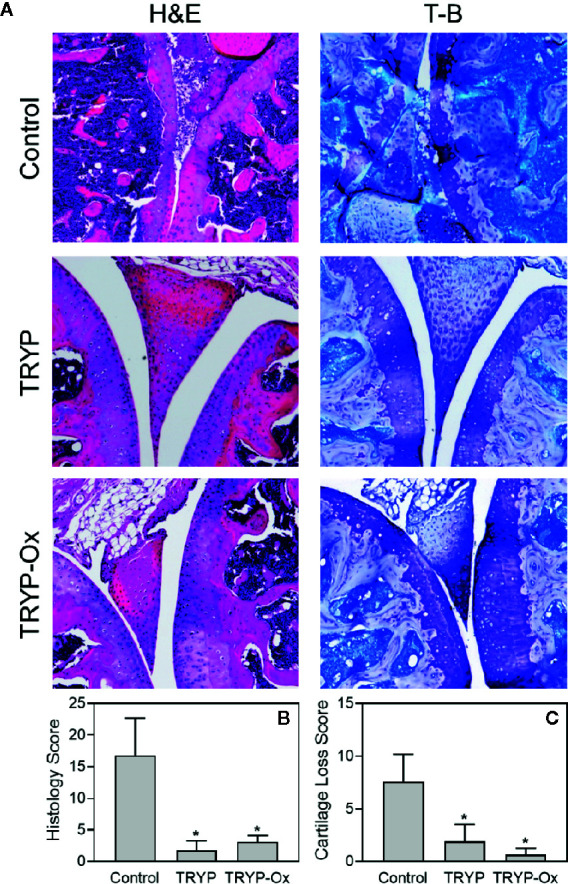
Effects of TRYP and TRYP-Ox on inflammation and cartilage loss in CIA mice. Starting at day 8 after CII challenge, mice were treated *i.p.* daily with 30 mg/kg TRYP or 30 mg/kg TRYP-Ox in sterile saline until day 42, when mice were euthanized. Control mice received saline alone. **(A)** Hematoxylin and eosin (H&E)- and toluidine blue (T-B)-stained knee sections. **(B, C)** Histology scores (0–3 per section) and cartilage loss scores (0–3 per section) were graded for each limb and knee section. The results show the mean of five mice per group ± S.D. *p < 0.05 compared with saline control.

Histopathological analysis was performed on ankle joints collected from CIA mice treated with TRYP or TRYP-Ox and compared with control, saline-treated mice. Representative images of stained joint tissue from mice treated with 30 mg/kg compounds are shown in **Figure 7A**. In contrast to the severe cartilage erosion, synovial hyperplasia, and infiltration of inflammatory cells observed in the joints of saline-treated CIA mice, there was little cartilage erosion, synovial hyperplasia, or cellular infiltration in the joints of TRYP and TRYP-Ox**-**treated CIA mice. Furthermore, histological scoring showed significant differences between treated and control groups (**Figures 7B, C** **)**, whereas the differences between TRYP- and TRYP-Ox-treated mice were not significant. Thus, consistent with the reductions in CIA clinical score, TRYP and TRYP-Ox treatment clearly protected these mice from cartilage destruction.

### TRYP and TRYP-Ox Decrease Serum Titer of CII-Specific Abs

**Figure 8:**
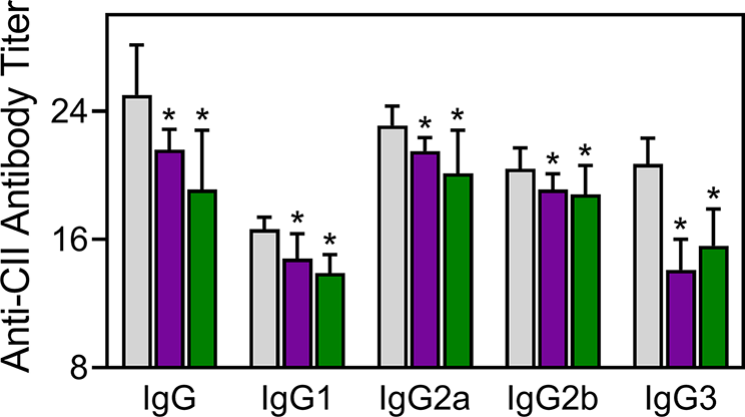
Effect of TRYP and TRYP-Ox on the antibody response to CII. Starting at day 8 after CII challenge, mice were treated *i.p.* daily with control saline (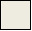), 30 mg/kg TRYP (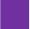), or 30 mg/kg TRYP-Ox (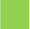) until day 42. Mice were euthanized, and sera were collected for measurement of anti-CII IgG, IgG1, IgG2a, IgG2b, and IgG3. The results show the mean of 10 mice per group ± S.D. *p < 0.05 compared with saline control.

Injected CII-specific Abs can induce transient arthritis, indicating that Ab responses are important in CIA pathogenesis (Myers et al., 1997; Nandakumar et al., 2003), and it has been shown that the anti-CII IgG antibody titer correlates with arthritis severity in CIA mice (Williams et al., 1998). We measured the levels of CII-specific Abs in TRYP-, TRYP-Ox-, and vehicle-treated CIA mice and found that the titers of the IgG, IgG1, IgG2a, IgG2b, and IgG3 were significantly lower in the compound**-**treated groups compared to those in the saline-treated group (**Figure 8**).

### TRYP and TRYP-Ox Decrease Proinflammatory Cytokine Levels in LN Cells

**Figure 9:**
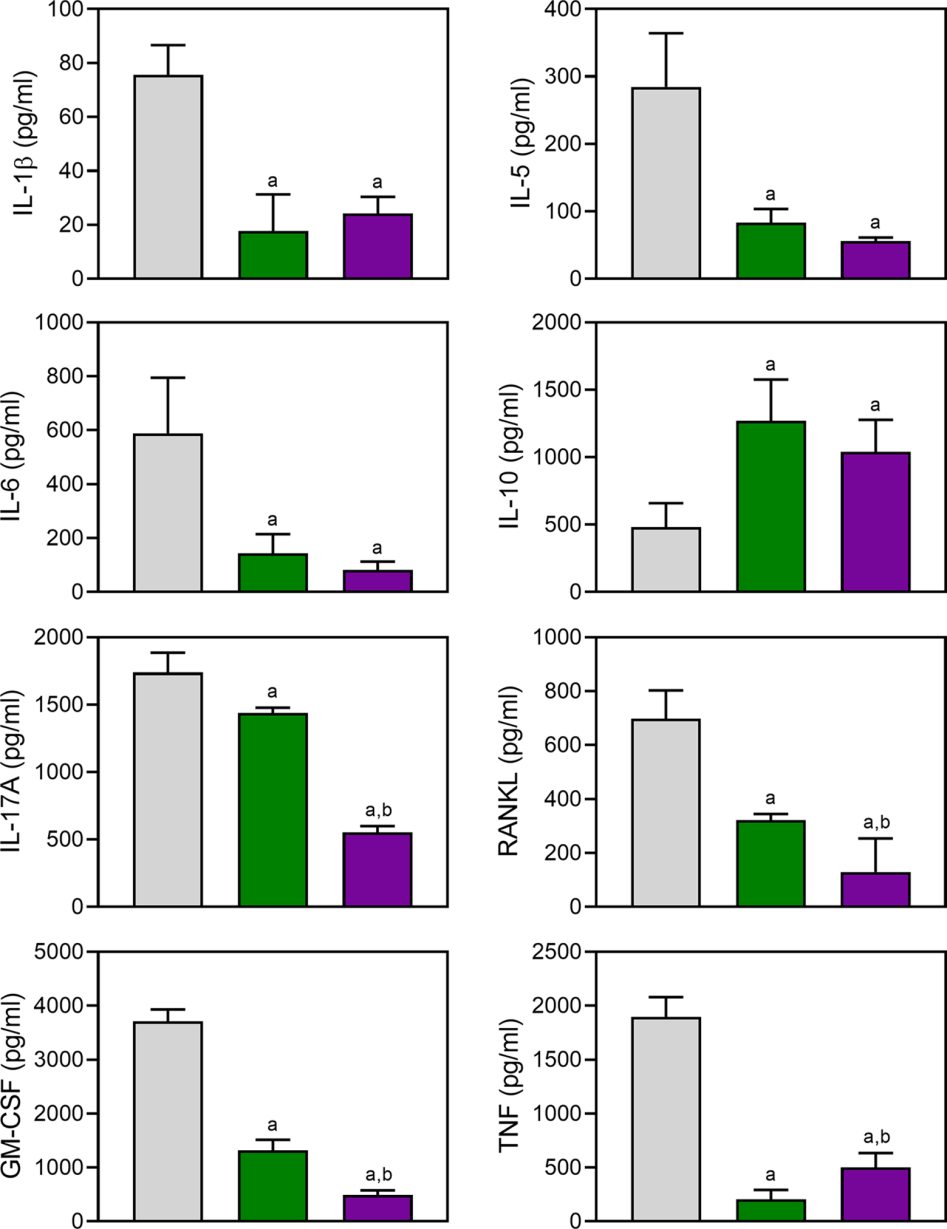
Effect of TRYP and TRYP-Ox on cytokine production by LN cells. Starting at day 8 after CII challenge, mice were treated *i.p.* daily with saline (0), 30 mg/kg TRYP, or 30 mg/kg TRYP-Ox until day 42. Mice were euthanized, and their LN mononuclear cells were purified and cultured for 3 days with CII. Cytokine levels were measured in supernatants by ELISA. The results show the mean of one experiment (three to four replicates) ± S.D., which is representative of three independent experiments. ^a^ *p *< 0.05 compared with the DMSO controls; ^b^ *p *< 0.05 compared with TRYP.

Proinflammatory cytokines produced by myeloid cells can facilitate inflammation, cartilage damage, and joint destruction in RA (Schurgers et al., 2011; Zhou et al., 2011; Azizi et al., 2013). To test whether TRYP and TRYP-Ox affected the production of inflammatory cytokines by CII-specific T cells, we evaluated CII-induced cytokine production by LN cells isolated from control and treated mice. We cultured the same numbers of cells from compound-treated and saline-treated CIA mice for 72 h in the presence of CII and found that the generation of proinflammatory cytokines (IL-1β, IL-5, IL-6, IL-17A, TNF, GM-CSF, and RANKL) was significantly reduced in compound-treated mice (30 mg/kg) compared to saline-treated CIA mice. In contrast, the production of the anti-inflammatory cytokine IL-10 was higher in compound-treated mice (**Figure 9**). Note that the levels of IL-17A, GM-CSF, and RANKL production by LN cells isolated from TRYP-Ox-treated mice were significantly lower compared to mice treated with TRYP.

### TRYP and TRYP-Ox Suppress CAIA Severity

**Figure 10:**
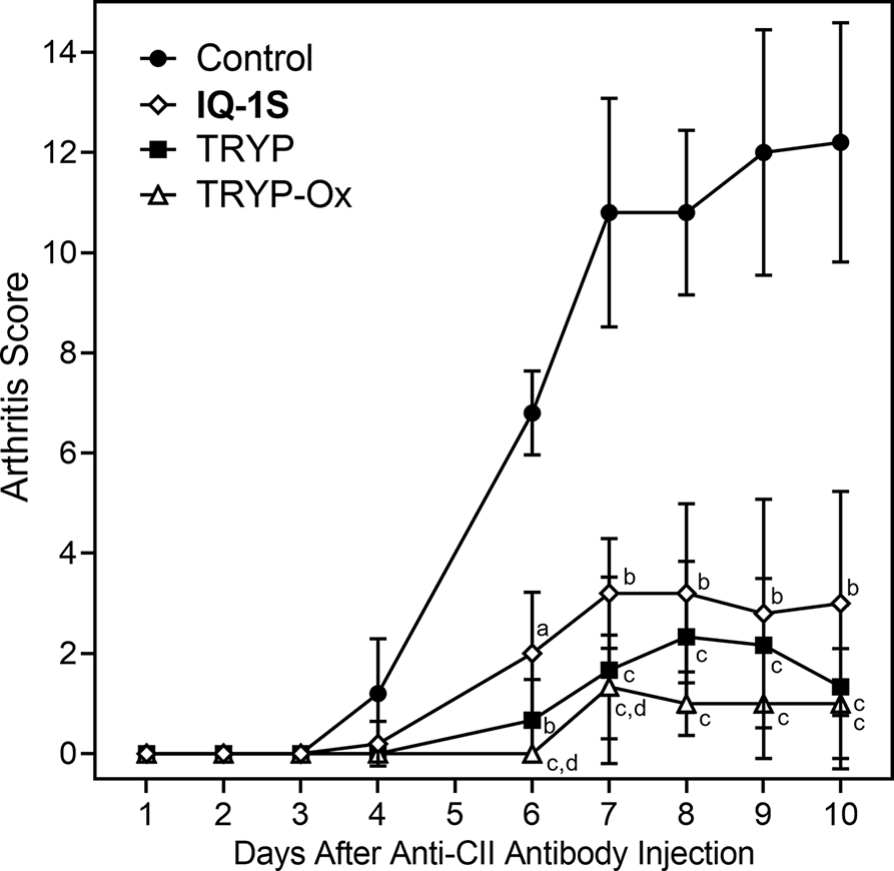
Therapeutic effects of TRYP and TRYP-Ox in CAIA mice. Starting at day 1 after the anti-CII antibody injection, mice were treated *i.p.* daily with control saline (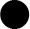), TRYP (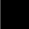), TRYP-Ox (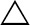), or **IQ-1S** (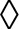) (30 mg/kg of each compound) until day 9, and clinical scores were determined. Results are shown as the mean of 5 mice per group ± S.D. ^a^ *p* < 0.05; ^b^ *p* < 0.01; ^c^ *p* < 0.001 compared with saline control, and ^d^ *p* < 0.05 compared with the **IQ-1S**–treated group.

The CAIA mouse model is another widely used animal model to screen for potential anti-RA compounds [for example (Chang et al., 2011)]. To evaluate the therapeutic potential of TRYP and TRYP-Ox in CAIA, treatment was initiated at day 1 after anti-CII antibody injection. We found that mice treated *i.p.* daily with 30 mg/kg TRYP or TRYP-Ox displayed significant reductions in the severity of CAIA compared with vehicle-treated mice, as assessed by the mean arthritis score (**Figure 10**). Although the differences in therapeutic effects of TRYP and TRYP-Ox were not significant, the therapeutic effects of TRYP and TRYP-Ox were significantly greater at days 6 and 7 compared to mice treated with **IQ-1S**, a JNK inhibitor that we previously showed to inhibit arthritis in the CIA model (Schepetkin et al., 2015).

**Figure 11:**
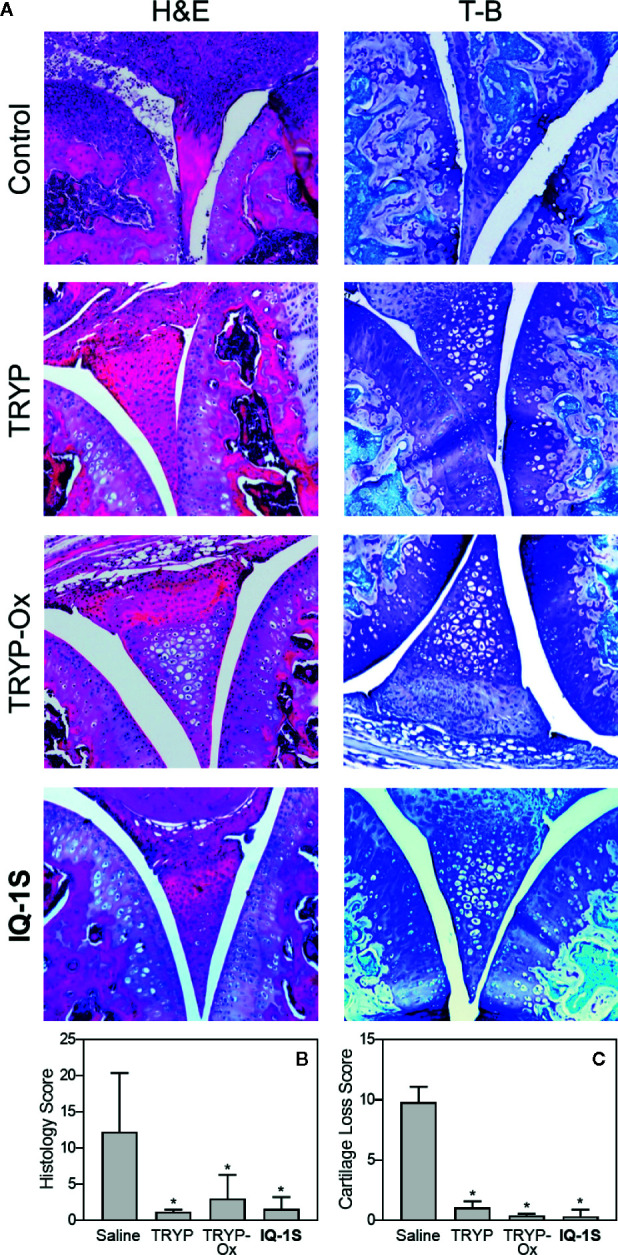
Effect of TRYP and TRYP-Ox on inflammation and cartilage loss in CAIA mice. Starting at day 1 after injection of anti-CII antibodies, mice were treated *i.p.* daily with 30 mg/kg of TRYP, TRYP-Ox, or **IQ-1S** in sterile saline until day 9. The mice were euthanized at day 14. Control mice received saline alone. **(A)** Hematoxylin and eosin (H&E)- and toluidine blue (T-B)-stained knee sections. **(B, C)** Histology scores (0–3 per section) and cartilage loss scores (0–3 per section) were graded for each limb and knee section. The results show the mean of five mice per group ± S.D. *p < 0.01 compared with saline controls.

Histopathological analysis was also performed on ankle joints collected from CAIA mice treated with 30 mg/kg TRYP or TRYP-Ox and compared to that of control mice (**Figure 11**). Severe cartilage erosion, synovial hyperplasia, and infiltration of inflammatory cells was seen in the joints of saline-treated CAIA mice. In contrast, there was little cartilage erosion, synovial hyperplasia, or cellular infiltration in TRYP and TRYP-Ox-treated CAIA mice. Furthermore, histological scoring showed significant differences between treated and control groups (**Figures 11B, C**). Thus, consistent with the reduced CAIA clinical score and the results using the CIA model, TRYP and TRYP-Ox treatment clearly protected CAIA mice from cartilage damage, as well as from severe joint inflammation.

## Discussion

RA is a chronic destructive autoimmune disorder that commonly leads to significant joint destruction that is mediated in part by proteases and cytokines produced by macrophages and FLS. Although current biological therapeutic strategies for RA have been effective in many cases, there are still issues of cost, loss of efficacy over time, toxicity, and protein degradation. Thus, new classes of therapeutics are needed that can be used alone or in combination with the current biologic RA treatments. In the present study, we identify TRYP-Ox and possibly TRYP as potentially novel RA therapeutics that could be pursued in drug development.

Previously, we demonstrated that TRYP-Ox had high affinity for JNKs and also inhibited pro-inflammatory cytokine production *in vitro* (Schepetkin et al., 2019), suggesting that it might be have potential in the development of treatments for inflammatory diseases, such as RA. To further evaluate potential targets of TRYP and TRYP-Ox, we performed a broad pharmacophore mapping screen using PharmMapper, which screens a large database of human protein targets. Screening of TRYP-Ox showed that JNK1, JNK3, and CFB were the top potential protein targets. These mapping results are supported by our previous kinase screening, which demonstrated that TRYP-Ox had affinity for all three JNKs, with the highest affinity for JNK1 (150 nM) and JNK3 (275 nM) (Schepetkin et al., 2019). Indeed, a number of studies have shown that JNKs play an important role in RA (Han et al., 2001; Bogoyevitch et al., 2010; Koch et al., 2015), as well as many other inflammatory diseases (Thalhamer et al., 2008; Nikoloudaki et al., 2020). CFB may also represent a therapeutic target in RA, as CFB deficient mice (CFB^-/-^) are highly resistant to CIA and CAIA and have a decreased CII-specific IgG antibody response in CIA (Hietala et al., 2002; Banda et al., 2006). Furthermore, local complement production, including CFB, may play a role in RA pathogenesis. Thus, the reduction local complement generation may represent a reasonable therapeutic approach for RA (Neumann et al., 2002).

In contrast to TRYP-Ox, the three top ranked targets for TRYP were AChE, CES-1, and TTR, which do not seem to be obvious targets associated with RA. Thus, it is interesting that TRYP was still fairly effective *in vivo* in treating CIA/CAIA. Although TTR has been reported to be significantly upregulated in the plasma of RA patients (Sharma et al., 2014), this protein has not yet been established as a pharmacological target for RA treatment. CES-1 may play important role in pharmacokinetics and pharmacodynamics of some anti-inflammatory drugs (Mishima et al., 1991), but CES-1 also has not been reported as a therapeutic target for RA. Recently rivastigmine, a known AChE inhibitor, was reported to have therapeutic effects in a complete Freund’s adjuvant-induced model of arthritis (Shafiey et al., 2018). Furthermore, it was found that AChE activity was increased with age and may increase RA risk and severity in elderly people (Sato et al., 2015). Therefore, AChE could be an additional co-target for agents with anti-arthritic activity. However, we found here that both TRYP and TRYP-Ox have relatively low anti-AChE effects, suggesting that AChE is probably not a therapeutic target for these compounds in CIA or CAIA. Note that TRYP has been reported to act on multiple signaling cascades associated with inflammation, including TNF/NF-κB and IL-6/STAT3 (Wang et al., 2018), leukotriene secretion (Pergola et al., 2012), and several cytokines required for TH17 polarization (Cheng et al., 2020). TRYP has also been reported to inhibit macrophage nitric oxide and prostaglandin E production upon exposure to oxidative stress (Ishihara et al., 2000), as well as inhibit cyclooxygenase-2 and indoleamine 2,3-dioxygenase activities (Danz et al., 2001; Pergola et al., 2012; Yang et al., 2013). Overall, the putative TRYP anti-inflammatory target associated with RA is still not clear and will require further investigation.

We demonstrate here for the first time that TRYP and TRYP-Ox may be promising therapeutics for treating RA, as shown by their ability to ameliorate clinical outcomes *in vivo* in CIA and CAIA mice, as well as reduce disease-associated anti-CII antibody production in CIA mice. CIA progression requires both adaptive and innate immune responses through the production of anti-CII-specific antibodies and the development of CII-specific T cells (Brand et al., 2003). We found that both TRYP and TRYP-Ox inhibited the production of anti-CII antibodies by B cells, especially IgG1 and IgG3, which represent T cell-dependent and T cell-independent responses, respectively (Stevens et al., 1988). Thus, therapeutic effects of TRYP and TRYP-Ox in CIA may be associated with direct inhibition of B cell activity or indirect effects on innate immune cells. Indeed, in both CIA and CAIA models, anti-CII antibodies have been reported to form immune complexes that activate myloid cell FcγR, leading to infiltration of monocyte/macrophages, neutrophils, and mast cells into the joints, where they release inflammatory mediators that initiate synovitis and bone destruction (Delire, 1994; Firestein and Corr, 2005).

Macrophages and FLS invade the synovium in RA and secrete proinflammatory cytokines and MMPs that enhance and sustain the inflammatory response (Kinne et al., 2000; Davignon et al., 2013). The primary cytokines required for inducing arthritis in autoimmune models are IL-1β, IL-6, IL-17A, TNF, GM-CSF, and RANKL (Nakae et al., 2003; Guma et al., 2010; Li et al., 2012; Tanaka, 2019). These cytokines play a key role in RA pathogenesis through their ability to recruit and stimulate macrophages, neutrophils, and mast cells (Drexler et al., 2008; Guma et al., 2010; Tanaka, 2019). Here, we found that TRYP and TRYP-Ox down-regulated IL-1β, IL-5, IL-6, IL17A, TNF, GM-CSF, and RANKL production by LN cells, while IL-10 production was increased. Levels of IL-17A, GM-CSF, and RANKL production by LN cells isolated from TRYP-Ox-treated mice were much lower compared to mice treated with TRYP. This finding may suggest JNK-dependent regulation of these cytokines. Indeed, RANKL and GM-CSF expression and(or) secretion in other cells, such as macrophages, osteocytes, and FLS, can be blocked by SP600125, another JNK inhibitor (Xu et al., 2015; Liu et al., 2016; Takeno et al., 2018). Among the different T cell subsets, a population of IL17-producing T helper cells (TH17) has been shown to be involved in model systems of autoimmunity (Weaver et al., 2007). Recently Cheng et al. (Cheng et al., 2020) reported that TRYP exhibits anti-TH17 activity through its ability to repress the expression of several cytokines required for TH17 polarization.

IL-1β plays important roles in inflammation and destruction of synovial tissue, cartilage, and bone in patients with RA (Tak and Bresnihan, 2000). IL-1β induces synoviolin, an E3 ubiquitin ligase, in synovial fibroblasts, which is involved in the overgrowth of synovial cells during RA (Gao et al., 2006). Therapeutic effects of some kinase inhibitors is associated with decreased IL-1β levels in the joints of CIA mice (McIntyre et al., 2003; Nishikawa et al., 2003). Here, we show that TRYP and TRYP-Ox significantly inhibited IL-1β–induced IL-6 secretion by SW982 synovial sarcoma cells and THP-1 monocytic cells, with TRYP-Ox being far more effective than TRYP.

The joint tissue damage associated with RA is due primarily to the aggressiveness of FLS in the synovial intima, and it is known that rheumatoid FLS produce MMPs that participate in extracellular matrix destruction in articular cartilage and cytokines that prolong inflammation (Burrage et al., 2006; Bartok and Firestein, 2010). Furthermore, JNK plays a key role in FLS *MMP* gene expression (Han et al., 1999; Liacini et al., 2002; Inoue et al., 2005; Kanai et al., 2020). In our studies, TRYP and TRYP-Ox inhibited IL-1β–induced MMP-1/3 secretion by HUVECs and SW982 cells and *MMP3* gene expression in FLS, with the most potent being TRYP-Ox in FLS and SW982 cells. Thus, part of the therapeutic effects of TRYP and TRYP-Ox may be due to their ability to target FLS and potentially reduce their aggressiveness in RA [e.g., (McIntyre et al., 2003; Nishikawa et al., 2003; Hegen et al., 2008)]. These results are consistent with our observations in CIA mouse model, where TRYP and TRYP-Ox decreased joint edema, cell migration, and cartilage erosion.

JNKs play important roles in many pathological processes, including autoimmune inflammatory disorders, such as RA (Bogoyevitch et al., 2004; Bogoyevitch et al., 2010; Mehan et al., 2011). A number of JNK inhibitors with anti-inflammatory properties have been developed (Bhagwat, 2007), yet few have been evaluated for treatment of arthritis. Previous studies demonstrated that SP600125 and **IQ-1S**, which are both JNK inhibitors, could reduce the development and pathogenesis of arthritis when evaluated in animal models (Han et al., 2001; Han et al., 2012; Schepetkin et al., 2015). Interestingly, the **IQ-1S** structure is related to TRYP-Ox, and, similarly to **IQ-1S**, TRYP-Ox was specific for JNK as compared to a variety of other kinases screened (Schepetkin et al., 2019). Moreover, TRYP-Ox is likely a prodrug for TRYP, because microsomal metabolism of aryl-oximes leads to the formation of ketone derivatives (Andronik-Lion et al., 1992). Although **IQ-18**, the ketone precursor of **IQ-1S**, did not have any anti-inflammatory activity or therapeutic effects in CIA (Schepetkin et al., 2012; Schepetkin et al., 2015), we show here that TRYP, the ketone precursor of TRYP-Ox, has therapeutic effects in both CIA and CAIA, although with lower efficacy. Thus, the therapeutic effects observed for TRYP-Ox *in vivo* are likely due to the combined effects of both TRYP-Ox and its metabolite TRYP.

In conclusion, we demonstrated that TRYP and TRYP-Ox have anti-inflammatory properties and potentially represent a novel class of quinazoline-based therapeutic agents that could be used in the development of treatment for RA. TRYP-Ox has additive beneficial therapeutic properties as a JNK inhibitor.

## Data Availability Statement

The raw data supporting the conclusions of this article will be made available by the authors, without undue reservation.

## Ethics Statement

The studies involving human participants were reviewed and approved by Institutional Review Board, University of California, San Diego School of Medicine, La Jolla, CA. The patients/participants provided their written informed consent to participate in this study. The animal study was reviewed and approved by Institutional Animal Care and Use Committee, Montana State University, Bozeman, MT 59717.

## Author Contributions

IS, LK, DH, AK, and MQ participated in research design. LK, IS, DH, AIK, and AK performed experiments. LK, IS, DH, AIK, AK, and MQ performed data analysis; IS, LK, DH, AIK, and MQ wrote or contributed to the writing of the manuscript.

## Funding

This research was supported in part by National Institutes of Health IDeA Program Grants GM110732, GM115371, and GM103474; USDA National Institute of Food and Agriculture Hatch project 1009546; the Montana State University Agricultural Experiment Station, and the Tomsk Polytechnic University Competitiveness Enhancement Program. Molecular modeling studies were supported by the Russian Science Foundation grant 17-15-01111.

## Conflict of Interest

The authors declare that the research was conducted in the absence of any commercial or financial relationships that could be construed as a potential conflict of interest.
